# Advance Directive Preferences: Comparing Medical-Social-Emotional vs. Medical-Based Approaches

**DOI:** 10.1093/geroni/igaf122.2600

**Published:** 2025-12-31

**Authors:** Ashley Shayya, Yuchi Young, Yufang Tu, Wan-Yu Chiu, Taylor Perre

**Affiliations:** Center for Human Services Research, University at Albany College of Integrated Health Sciences, Albany, New York, United States; University at Albany College of Integrated Health Sciences, Albany, New York, United States; North Dakota State University, Fargo, North Dakota, United States; National Taiwan University, Zhongzheng District, Taipei, Taiwan (Republic of China); Home Care Association of New York State, Albany, New York, United States

## Abstract

This study explores adults’ preferences for and recommendations to improve medical-based (POLST/MOLST) and medical-social-emotional-based (Five Wishes) advance directives, with a focus on age-related differences. US community-dwelling adults completed a survey on advance directives. Univariate analyses were used to assess advance directive type preferences, bivariate analyses evaluated age differences, and a thematic analysis identified recommendations for improvement. Participants, particularly young adults, preferred the Five Wishes form over the POLST/MOLST. Three key themes emerged from participants’ recommendations for improving the advance directives: content, formatting, and no recommendations. A greater proportion of participants shared content recommendations for the POLST/MOLST and formatting recommendations or no recommendations for the Five Wishes form. For the POLST/MOLST, adults emphasized the need for more detailed descriptions, less medical jargon, less dense formatting, and an overall more holistic approach to end-of-life care. For the Five Wishes form, adults recommended shortening the form, adding more medical treatment questions, and altering the format of the wishes to be more user friendly. These recommendations to improve the POLST/MOLST and Five Wishes form may be useful for advance directive creators and policymakers to consider when making future adaptations to these advance directives. Preferences for medical-social-emotional-based and medical-based advance directives should be considered by medical practitioners, policymakers, and public health professionals when promoting advance directives, especially among young adults.

